# Genomics Approach of the Natural Product Pharmacology for High Impact Diseases

**DOI:** 10.1155/2018/9468912

**Published:** 2018-04-08

**Authors:** Yong Wang, Qin Feng, Peijian He, Lixin Zhu, Guoxun Chen

**Affiliations:** ^1^Lifescience School, Beijing University of Chinese Medicine, Beijing, China; ^2^Institute of Liver Disease, Shuguang Hospital, Shanghai University of Traditional Chinese Medicine, Shanghai, China; ^3^Department of Medicine, Emory University, Atlanta, GA, USA; ^4^Digestive Diseases and Nutrition Center, Women and Children's Hospital of Buffalo, Department of Pediatrics, the State University of New York at Buffalo, Buffalo, NY, USA; ^5^Department of Nutrition, University of Tennessee, Knoxville, TN, USA

According to the World Health Organization, high-impact diseases (HID) including cardiovascular diseases, metabolic diseases, and a variety of types of cancer are the top challenge to the entire medical community globally [1]. Therapeutic strategies using bioactive compounds have been extensively investigated as natural products have become a rich source for drug discovery due to their structural diversities and a wide range of pharmacophores. However, despite considerable gains in the pharmacological research of natural products, their applications are still limited, partly because of the technical barriers to comprehensively understand their molecular targets and pharmacological mechanisms [2].

Recently, omics technologies including genomics, proteomics, transcriptome, and metabolomics have been developed and improved significantly. This is especially true for RNA sequencing together with the system-based pharmacology, which has overcome the barriers and greatly improved our understanding of the pharmacology of natural products for the prevention and treatments of HID.

For example, Shuanglong Formula (SLF) and Qishen granules (QSG) are well-studied natural products for the treatment of cardiovascular diseases. Multiomics including metabolomics and comparative proteomics were performed to investigate the SLF's effects on the regulation of myocardial energy metabolism [3, 4]. Y. Wang et al. elucidated that QSG used in the treatment of heart failure concurrently attenuate inflammation and NO production through gene expression analysis with a novel strategy called keystone gene-based group significance analysis [5]. Moreover, by integrating the proteomic approach and bioinformatics methods, L.-X. Feng et al. demonstrated that salvianolic acid B (SB), one component in QSG, impacted the signal cascade from the epidermal growth factor receptor to heat shock protein 27 (HSP27) and mitochondria [6]. Similarly, using a combined approach of transcriptome and microbiome analyses, Q. Feng et al. found that Qushi Huayu Decoction simultaneously activated the hepatic antioxidative mechanism, suppressed the hepatic lipid synthesis, and amplified the regulatory T cell-inducing microbiota in the gut [7].

In addition, new omics approaches are also developed to identify the active indigents and potential targets of natural products. A novel approach for relative and absolute quantitation was established to specifically and comprehensively identify the protein targets of andrographolide (Andro) [8], and BATMAN-TCM, a bioinformatics tool of network pharmacology, has been available online for predicting the targets and pharmacologic pathways of natural products [9].

Due to the diverse components of natural products, systematically integrating the readouts of genomics, transcriptomics, proteomics, and metabolomics will dramatically facilitate our understanding of molecular mechanisms of natural product actions, as well as identifying biomarkers or targets for future research and clinical practice. Recently, an integrated proteomic and metabolomics research demonstrated that a combination of natural products comprising of *Rheum palmatum L.*, *Gardenia jasminoides* Ellis, and *Artemisia annua L.*, had an improved therapeutic effect on hepatic injury syndrome through regulating dynamic patterns in metabolic biomarkers and target proteins and activating both intrinsic and extrinsic pathways [10].

Unsurprisingly, more omics studies are currently under way to reveal the molecular mechanisms of treatments using natural products. The current issue is a collection of selected works that use the approaches of genomics, metabolomics, RNA sequencing, and network pharmacology to comprehensively elucidate the natural product pharmacology in the treatments of HID including heart failure, diabetes and its complications, nonalcoholic fatty liver disease, and depression. It is a timely update to our knowledge on both the molecular mechanism and novel drug discovery for HID.

*Yong Wang* *Qin Feng* *Peijian He* *Lixin Zhu* *Guoxun Chen*
